# Pathogenicity and Complete Genome Characterization of Fowl Adenoviruses Isolated from Chickens Associated with Inclusion Body Hepatitis and Hydropericardium Syndrome in China

**DOI:** 10.1371/journal.pone.0133073

**Published:** 2015-07-13

**Authors:** Jing Zhao, Qi Zhong, Ye Zhao, Yan-xin Hu, Guo-zhong Zhang

**Affiliations:** Key Laboratory of Animal Epidemiology and Zoonosis, Ministry of Agriculture, College of Veterinary Medicine, China Agricultural University, Beijing, People’s Republic of China; The University of Melbourne, AUSTRALIA

## Abstract

In this study, we determined and genetically characterized three fowl adenoviruses isolated from chickens with inclusion body hepatitis (IBH) and hydropericardium syndrome (HPS) in China and assessed their pathogenicity. The full genome of HBQ12, BJH13 and JSJ13 was found to be 44,081, 43,966 and 43,756 nucleotides long, respectively. Sequence alignment and phylogenetic analysis revealed that strain HBQ12 and BJH13 were clustered together belonging to fowl adenoviruses D species and serotyped as FAdV-11, whereas strain JSJ13 was classified into fowl adenoviruses C species and serotyped as FAdV-4. To our knowledge, this is the first report of FAdV-4 strain circulating in China. The pathogenicity test showed that mortality for chickens infected with HBQ12 and JSJ13 within 21 days post infection (dpi) was 8.6% and 28.6%, respectively. Necropsy displayed mild or severe hepatitis and hydropericardium at 3 and 5 dpi as well as dead chickens. Viral DNA was detected in almost all tissues sampled from dead chickens. These results revealed that fowl adenovirus strains HBQ12 and JSJ13 are capable of causing IBH and HPS in chickens, indicating that preventive measures against FAdV infection on poultry farms should be implemented in China.

## Introduction

Adenoviruses (AdVs) are non-enveloped double stranded DNA-viruses, which belong to the family *Adenoviridae*. The family *Adenoviridae* is currently divided into five genera: *Mastadenovirus*, *Aviadenovirus*, *Atadenovirus*, *Siadenovirus*, and *Ichtadenovirus* [1, 2]. Chickens can be infected by fowl adenoviruses (FAdVs), belonging to the genus *Aviadenovirus*, egg drop syndrome (EDS) virus (duck adenovirus A, genus *Atadenovirus*) and turkey hemorrhagic enteritis (HE) virus (turkey adenovirus A, genus *Siadenovirus*).

FAdVs have a worldwide distribution and have been grouped into 5 species (FAdV-A to FAdV-E) with 12 serotypes (FAdV-1 to 8a and -8b to 11) based on restriction enzyme digest pattern and serum cross-neutralization test [3]. The most notable diseases associated with FAdVs-infection in chicken are the inclusion body hepatitis (IBH), the hydropericardium syndrome (HPS) and the gizzard erosions (GE) [4–6]. All 12 serotypes of FAdVs have been associated with outbreaks of IBH. Classical IBH affects mostly 3–5 weeks old chickens and is characterized by mortality approaching 10% and hepatic necrosis with microscopic eosinophilic or basophilic intranuclear inclusion bodies in hepatocytes [7, 8]. Most HPS are caused by FAdV serotype 4 (FAdV-4), characterized by accumulation of transparent or straw-colored fluid in the pericardial sac, nephritis, and hepatitis with a high mortality of 30–70% [9, 10]. GE is also induced by several serotypes of FAdVs and frequently found in slaughtered broiler chickens [11–13].

In recent years, the clinical cases of IBH and HPS have been increasing all over the world, resulting in considerable economic losses in many countries, such as USA [14], India [15], Canada [8], Hungary [16], Korea [17] and Japan [7]. A few confirmed outbreaks of IBH have been previously reported in China [18]. However, the number of IBH or HPS cases submitted to and diagnosed by the Diagnostic Center of Livestock and Poultry Epidemic Diseases at China Agricultural University has been increasing since 2012. Most cases were observed in broilers of 3 to 5 weeks of age, and occasionally in layers and breeder pullets aged 10 to 20 weeks. Mortality reported during these outbreaks varied from a slight increase to over 30% in severe cases and FAdVs were isolated from all the cases, but the molecular and pathogenic characterization of these viruses involved in IBH or HPS outbreaks were still unknown.

In the present study, three FAdV strains isolated from recent outbreaks which showed higher mortality rate in the affected flocks were characterized by phylogenetic assays and pathogenic analysis. We determined the complete nucleotide sequence of viral genome, investigated their genomic organization and relationship with other FAdVs, and selected two different isolates for further observation of their pathogenicity in SPF chickens.

## Materials and Methods

### Animal and ethics statement

One hundred and five 3-week-old specific-pathogen-free (SPF) chickens were used to determine the pathogenicity of two FAdV strains. All chickens were kept in isolators at China Agricultural University throughout the experiment and the rearing facilities were approved by Beijing Administration Committee of Laboratory Animals under the leadership of the Beijing Association for Science and Technology, the approval ID is XYXK (Jing) 2013–0013. The study was carried out in strict accordance with the Guidance for the Care and Use of Laboratory Animals formulated by the Ministry of Science and Technology of the People's Republic of China. The protocol (including the possibility of animal death without euthanasia) was specifically considered and approved by the Animal Welfare and Ethical Censor Committee at China Agricultural University. All surgery was performed under sodium pentobarbital anesthesia, and all efforts were made to minimize suffering.

### Origin of the strains

Three FAdV isolates, designated as HBQ12, BJH13 and JSJ13 respectively, were isolated from different geographical areas of China in 2012–2013 from livers of IBH-affected birds which presented with an accumulation of near-amber fluid in the pericardial sac and an enlarged discolored liver with foci of hemorrhage and/or necrosis. The isolates were further identified by polymerase chain reaction (PCR) with a pair of primers (forward: TGCTCGTTGTGGATGGTGAA; reverse: CTCCGTGTTGGGCTGGTC) based on the polymerase gene of the available FAdV nucleotide sequences strain A-2A (GenBank Accession No. AF083975), which specifically amplify a 564-bp fragment of FAdVs. Reactions were performed according to the following protocol: 95°C for 5 min, followed by 30 cycles of 95°C for 45 s, 58°C for 45 s, 72°C for 45 s, and a final elongation step of 10 min at 72°C. PCR products were analyzed by electrophoresis.

The isolates were passaged three times in SPF embryonated chicken eggs via the chorioallantoic membrane (CAM) route following standard method [19]. The CAM from infected embryos was harvested and homogenized up to 10% (w/v) in 0.01 M phosphate-buffered saline (pH 7.4), and clarified by low-speed centrifugation. The supernatant was passed through a 0.45-μm filter. Titration of the virus was carried out by inoculating serial ten-fold dilutions of CAM homogenate into 10-day-old SPF embryonated chicken eggs via the CAM. The median embryo infective dose (EID50) of the FAdV isolate was analyzed using the formula of Reed and Muench [20]. Infected CAM homogenate with a titer of >10^3.0^ EID_50_/0.2 ml was used as the source of virus for the infection.

### Primer design and DNA extraction

Based on the available FAdV nucleotide sequences strain A-2A (FAdV-D, GenBank Accession No. AF083975) and ON1 (FAdV-C, GenBank Accession No. GU188428), specific primers were designed to amplify the complete genome sequences of HBQ12, BJH13 and JSJ13, as shown in S1 and S2 Tables. All the primers were synthetized by Sangon Biotech (Shanghai, China). Viral DNAs from infected CAM homogenates were extracted using the DNAVzol (Vigorous, Beijing, China) according to the manufacturer's instructions and then used for the PCR amplification.

### PCR and sequencing

PCR amplification was performed in a thermocycler (Biometra, Goettingen, Germany) using the designed specific primers. Briely, 50 μl PCR reaction mixture containing 1μl (10 pmol) of each primer, 25μl Taq SuperMix (TransGen, Beijing, China), 4μl DNA and 19μl nuclease free water was prepared. Reactions were performed according to the following protocol: 95°C for 5 min, followed by 30 cycles of 95°C for 45 s, 53°C to 60°C for 45 s, 72°C for 1.5 min, and a final elongation step of 10 min at 72°C. PCR products were examined by electrophoresis on a 1.0% (w/v) agarose gel and visualized after Goldview staining. PCR products of the expected length were sequenced directly or purified with a Gel Extraction kit (Omega, Norcross, GA, USA), then cloned into the pEASY-T1 cloning vector (TransGen, Beijing, China) according to the manufacturer’s instructions and sequenced at BGI (Beijing, China). The sequencing was performed using the Sanger dideoxy sequencing method with an ABI 3730XL automatic sequencing apparatus. Both strands of the cloned DNA fragments were sequenced and discrepancies between sequences of the two strands were resolved by repeated reactions until a consensus was achieved. The complete sequences of three FAdV isolates were manually assembled using Seqman program in the DNAstar software package (version 5.01, DNAstar, Madison, WI, USA).

### Sequence alignment and phylogenetic analysis

The complete genome sequences of the three FAdV isolates were aligned with other available FAdV genome sequences in the GenBank database to determine the nucleotide sequence homologies using the ClustalW multiple alignment algorithm in the MegAlign program of the DNAstar software suite (version 5.01, DNAstar, Madison, WI, USA). A phylogenetic tree was constructed using MEGA4.1 software (Molecular Evolutionary Genetics Analysis, version 4.1) by Neighbor-Joining method (1000 replicates for bootstrap). The evolutionary distances were computed by Pairwise Distance method using the Maximum Composite Likelihood Model [21].

### Pathogenicity assessment of two FAdV isolates in SPF chickens

One hundred and five 3-week-old SPF chickens were used to determine the pathogenic potential of two FAdVs. The chickens were randomly divided into three groups, with 35 birds in each group. Chickens were inoculated with either 10^3.5^ EID_50_ of strain HBQ12 or JSJ13 via an oral route. The control birds received the same volume of phosphate-buffered saline (PBS). All birds were monitored twice daily and scored for clinical signs for 21 days. Clinical signs were given daily clinical scores: 0 for normal, 1 for mild depression, 2 for severe depressed, 3 for paralysis/prostration, and 4 for death. At 3 and 5 days post-infection (dpi), 4 chickens from each group were euthanized with intravenous injection of sodium pentobarbital (200 mg/ml), and heart, liver, spleen, lung, kidney, bursa, cecal tonsil, proventriculus, pancreas, thymus, trachea and small intestine were collected. Some birds died between the intervals of clinical observation and same tissue samples were collected. Tissue samples were placed into 10% formalin for histology or stored at -70°C for DNA detection using PCR method. In addition, 10 serum samples per group collected at 14 and 21 dpi were tested for FAdV-specific antibodies (Abs) by a commercial enzyme-linked immunosorbent assay (ELISA) kit (BioChek, Scarborough, ME, USA).

### Viral DNA detection of tissue samples

Collected tissue samples were used to determine tissue distribution of the virus by PCR. Total DNA was extracted from 200 μL of the homogenized tissue samples with DNAVzol Reagent (Vigorous, Beijing, China) according to manufacturer’s instructions. PCR was performed as described in section “Origin of the strains”.

### Histopathology

Samples of liver, kidney, proventriculus and small intestine were fixed in 10% neutral formalin for 48h at room temperature. Tissues were then routinely processed, embedded in paraffin wax, and cut into 5-μm sections. The sections were stained with hematoxylin and eosin (H&E) and examined for lesions associated with FAdV infection using light microscopy. The following scoring scheme was used to assess histopathological lesions in sampled tissues: 0 for no lesion, 1–3 for mild lesions, 4–6 for morderate lesions and 7–10 for severe lesions.

### Immunohistochemistry

FAdV antigen was detected in liver by immunohistochemical staining. In brief, 5-μm liver tissue sections were incubated in 10% normal goat serum in PBS for 30 min to block nonspecific binding sites. Slides were further incubated with chicken anti-FAdV hyperimmune serum (1:1000 dilution in 0.01 M PBS, Tiantech, Beijing, China) for 1 h, followed by incubation with a horseradish peroxidase-conjugated rabbit anti-chicken IgG (Solarbio, Beijing, China) for 1 h. The reaction was visualized by the addition of 3,3-diaminobenzidine (Sigma, St. Louis, MO, USA) for 15 min. The sections were counterstained with hematoxylin, air dried, and examined by light microscopy. The numbers of positive cells were quantitatively analyzed under a light microscope at ×40 magnification.

### Statistical analysis

Statistical analysis was performed by two-way analysis of variance (ANOVA) contained in the Prism 5.0 software package (GraphPad software Inc., San Diego, CA, USA), and a *P* value of <0.05 was considered statistically significant. Results are expressed as means and standard deviations.

## Results

### Genome size and organization of three FAdV isolates

**Fig 1:**
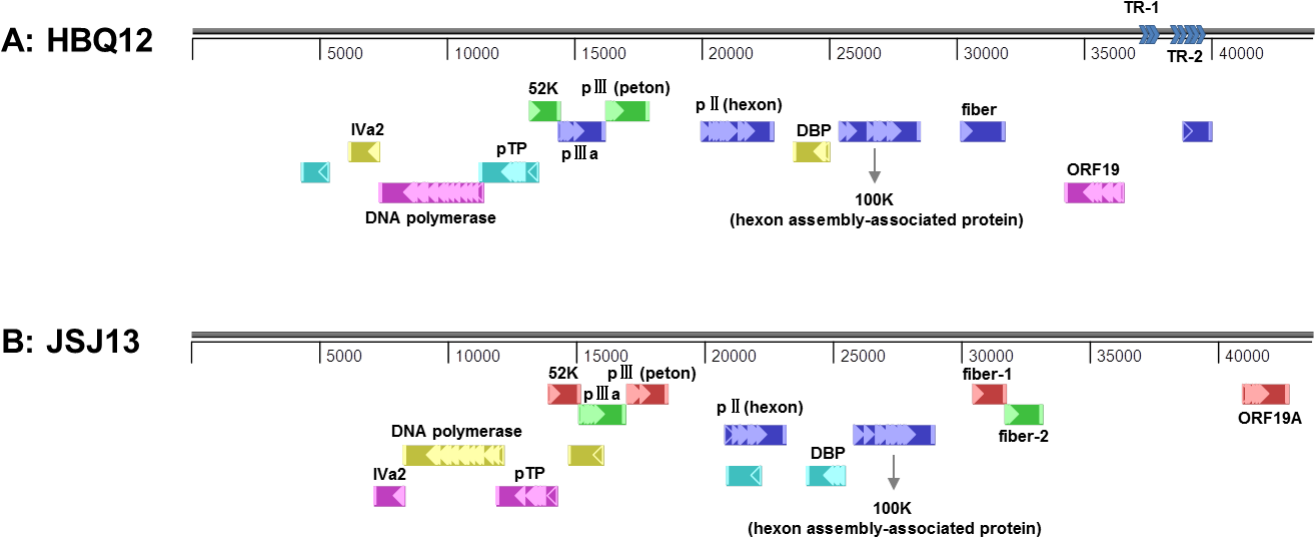
Schematic representation of HBQ12 and JSJ13 genomes, members of the genus Aviadenovirus.

The whole genome nucleotide sequences of HBQ12, BJH13 and JSJ13 isolates are available in the GenBank database under the accession numbers KM096545, KM096546 and KM096544, respectively. The full genome for HBQ12, BJH13 and JSJ13 was found to be 44,081, 43,966 and 43,756 base pairs (bp) in length, respectively. The strain HBQ12 genome contained two regions of repeated sequences TR-1 (the shorter repeat region) and TR-2 (the longer repeat region), as shown in Fig 1. Unlike other FAdV-D strains, TR-1 of HBQ12 contained three identical and contiguous 33 bp-direct repeats. The exact nucleotide sequence of TR-2 was found to consist of 5 identical and contiguous, 135 bp long direct repeats. Strain BJH13 only contained TR-2 and JSJ13 isolate had no repeated sequences of TR-1 or TR-2. Both inverted terminal repeat (ITR) sequences in strain HBQ12 and BJH13 were 72 bp, while in JSJ13 isolate, it is 56 bp in length.

### Sequence alignment and analysis

The percent sequence identity for available aviadenovirus whole genomes are given in Table 1. Strain HBQ12 and BJH13 were almost identical (99.7%) at the nucleotide level and they showed the highest sequence homology (95.8%) with strain A-2A (FAdV-D, Accession No.AF083975) isolated in the US at nucleotide level. Whereas they showed a low sequence identity (< 61.0%) with the members of other aviadenovirus species. JSJ13 strain was more matched to the KR-5 strain (FAdV-C) isolated in Japan (Accession No. HE608152, 98.4% of identity at the nucleotide level). Sequence identities between JSJ 13 and other species ranged from 38.6% (between JSJ13 and FAdV-A) to 49.7% (between JSJ13 and FAdV-E).

**Table 1:** Percent nucleotide sequence identities of the whole genomes of aviadenoviruses.^a^

Species	Strain	Accession number	Homology		
			HBQ12	BJH13	JSJ13
FAdV-A	CELO	U46933	39.2	39.3	38.7
FAdV-B	340	NC_021221	60.7	61.0	39.9
FAdV-C	ON1	GU188428	39.0	39.1	**98.0**
FAdV-C	KR-5	HE608152	39.1	39.1	**98.4**
FAdV-D	A-2A	AF083975	**95.8**	**95.8**	39.1
FAdV-E	HG	GU734104	56.2	58.1	49.7
FAdV-D	HBQ12	KM096545	/	100	39.4
FAdV-D	BJH13	KM096546	100	/	39.3
FAdV-C	JSJ13	KM096544	39.4	39.3	/

^a^Alignment was performed by using ClustalW. Strain HBQ12, BJH13 and JSJ13 were sequenced in this study and other aviadenovirus strains were obtained from the GenBank database.

### Phylogenetic analysis of three FAdV strains

**Fig 2:**
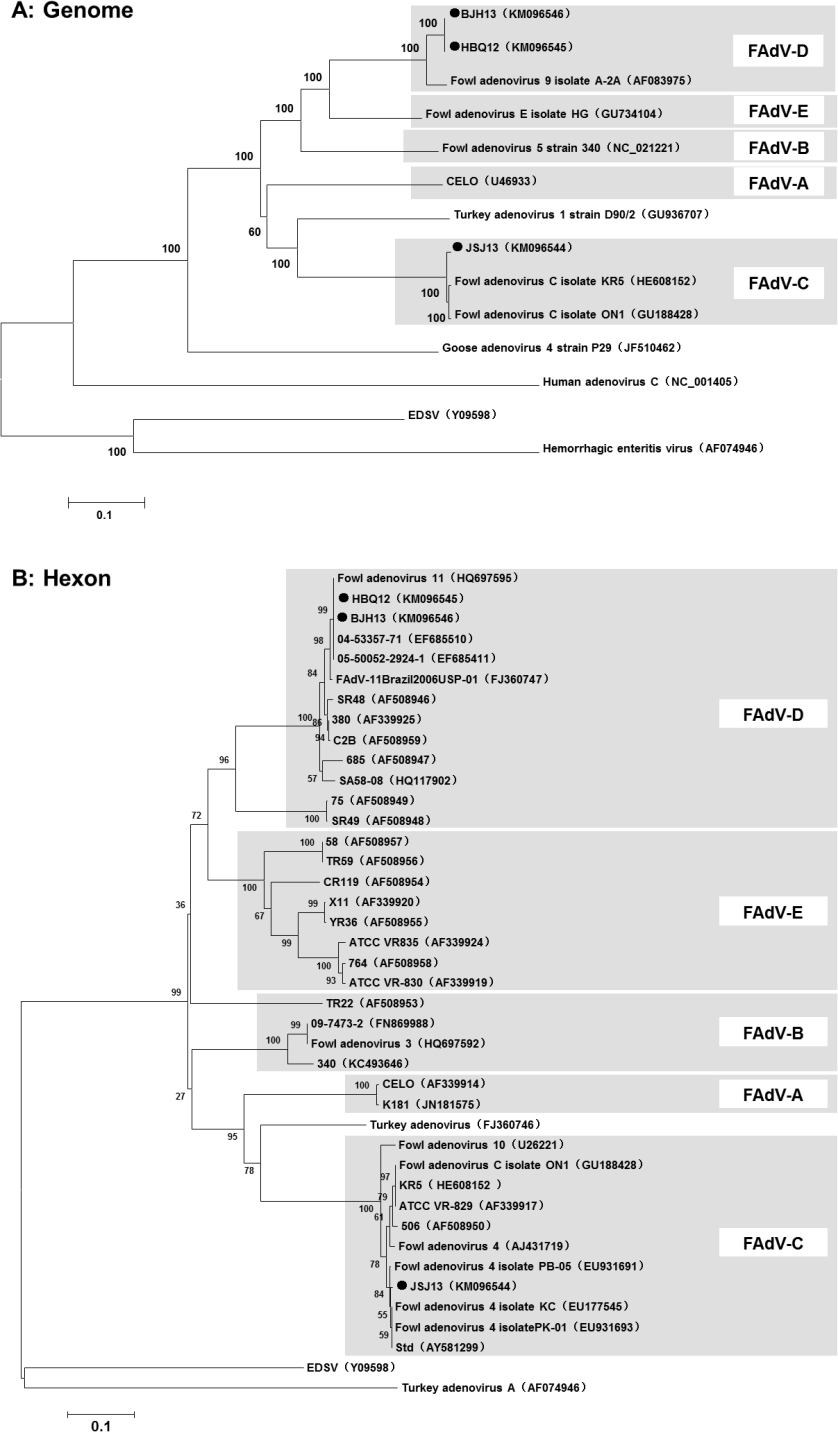
Phylogenetic tree based on the complete genome (A) and hexon gene (B) sequences of avian adenoviruses belonging to genera Aviadenovirus. Strains HBQ12, BJH13 and JSJ13 were sequenced in this study, other avian adenovirus strains were previously sequenced. Phylogenetic tree was generated by the using Neighbor-Joining method in the MEGA4.1 software. Bootstrap values were indicated for the major nodes. Accession numbers of already published adenovirus sequences were given in the brackets.

According to phylogenetic analysis based on the complete genome (Fig 2A), strain HBQ12 and BJH13 were classified into the same cluster. Both of them belonged to FAdV-D and had a close genetic relationship with strain A-2A (GenBank Accession No. AF083975). Strain JSJ13 was classified into the same cluster (FAdV-C) with ON1 (GenBank Accession No. GU188428), which was reported as the reference strain of serotype 4 of FAdV. The similar evolutionary relationships were obtained from the phylogenetic tree based on the hexon gene (Fig 2B).

### Clinical signs and gross pathology

**Fig 3:**
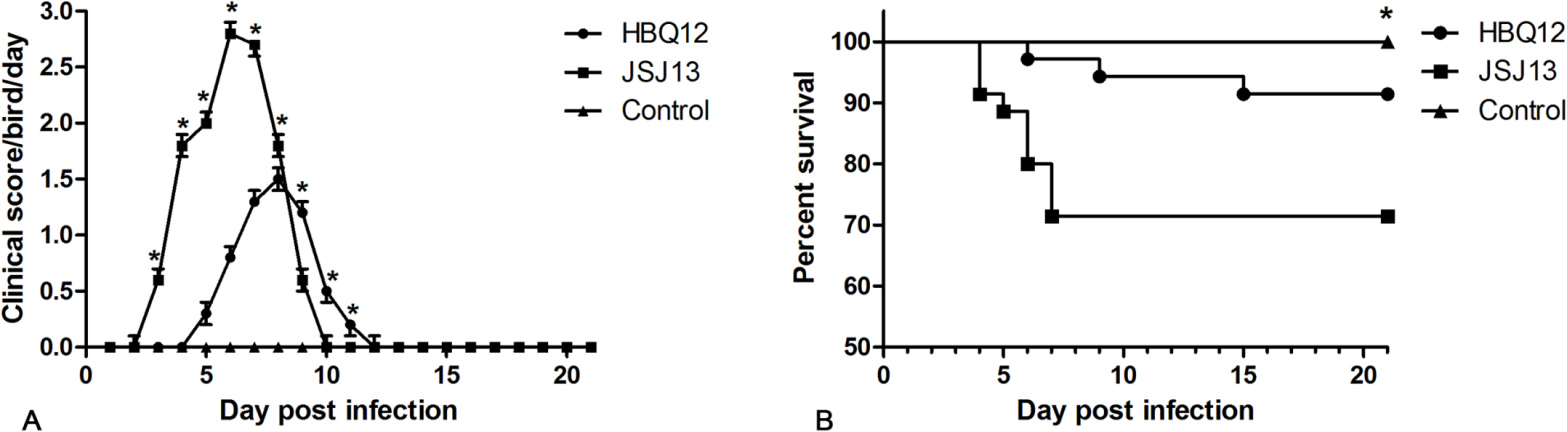
Clinical scores (A) and survival rates (B) of chickens after inoculation with FAdV HBQ12 or JSJ13 strain. Clinical scoring: 0 for normal, 1 for mild depression, 2 for severe depressed, 3 for paralysis/prostration, and 4 for death. The mean scores per group per day are shown. The error bars indicate standard deviations. Asterisks (*) mark the days in which the clinical scores were significantly different between groups (P <0.05). The percentage of birds that survived in the infected groups were significantly lower than in the control group (P <0.05, log rank analysis).

**Fig 4:**
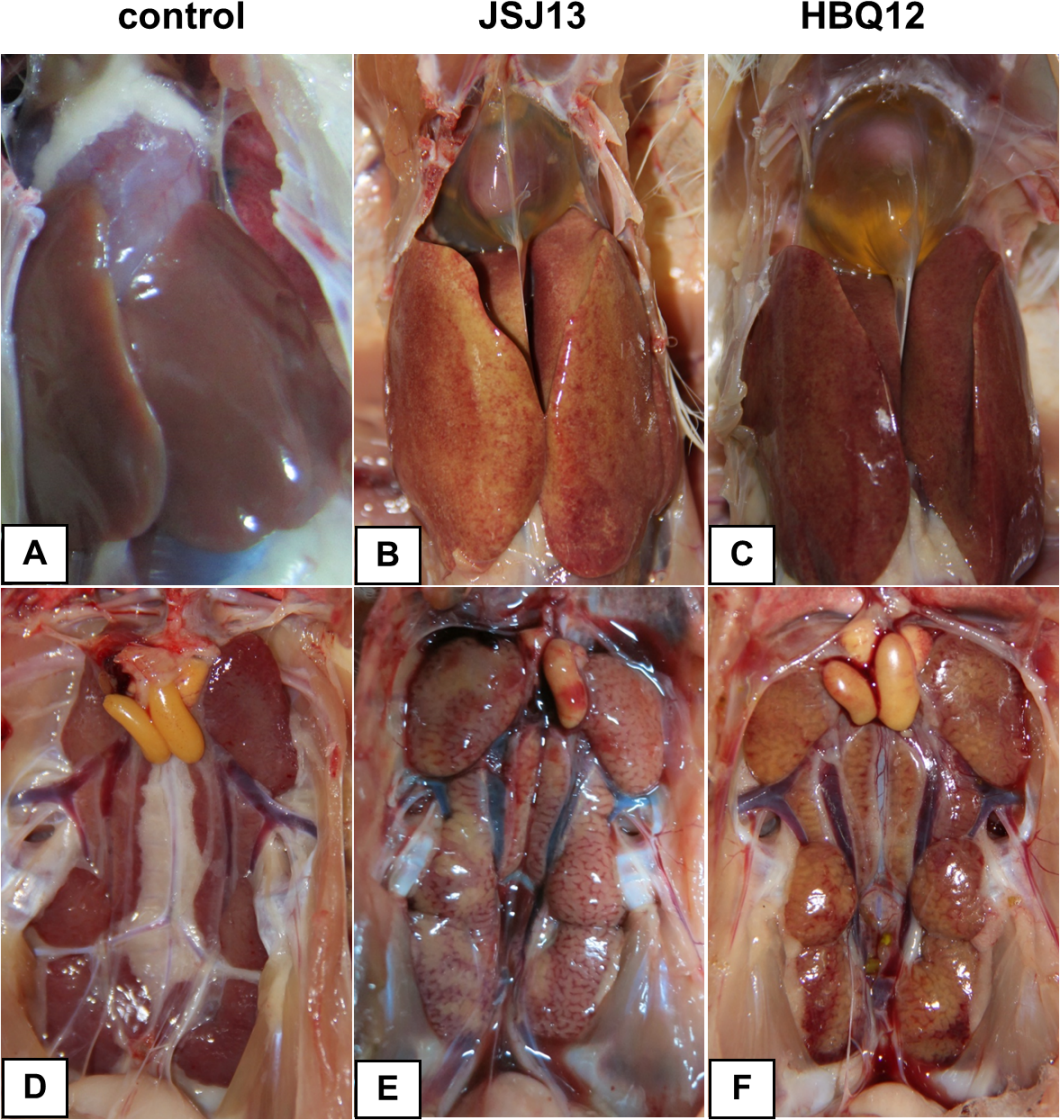
Gross lesions in liver, heart, and kidney from dead chickens at 6 dpi. (B&C) Swollen and friable liver with multifocal areas of necrosis and petechial haemorrhage and hydropericardium. (E&F) Mild or severe enlargement in the kidney of the infected chickens.

Chickens inoculated with 10^3.5^EID_50_ of HBQ12 or JSJ13 strain showed depression between 3 and 15 dpi. Flight reactions were significantly reduced. Mortality commenced from 4 dpi and continued for 6 days. During the experimental period, different clinical scores and mortality rates were observed in HBQ12- and JSJ13- infected animals. The clinical scores for HBQ12 infection were less than those of JSJ13 infection at 3 to 8 dpi (P<0.05) (Fig 3A). While 3 chicks died as a result of HBQ12 infection, 10 chicks died as a result of JSJ13 infection, and the survival rates were 91.4% and 71.4%, respectively, the survival rate showed significant difference between the JSJ13-infected group and the control group at the end of observation period (P<0.05), as shown in Fig 3B. At 3 and 5 days-post-inoculation, mild or severe hepatitis and hydropericardium were observed in necropsy. The pathological characteristics of all dead birds had a high similarity. The major post mortem findings were the swollen, yellow brown coloured liver with necrotic foci and the flabby heart with a severe hydropericardium (Fig 4B and 4C). The swollen and pale kidneys were also observed in most dead birds (Fig 4E and 4F). No significant gross lesions were present in the counterpart tissues of uninoculated control chickens (Fig 4A and 4D).

### Histology and immunohistochemistry

**Fig 5:**
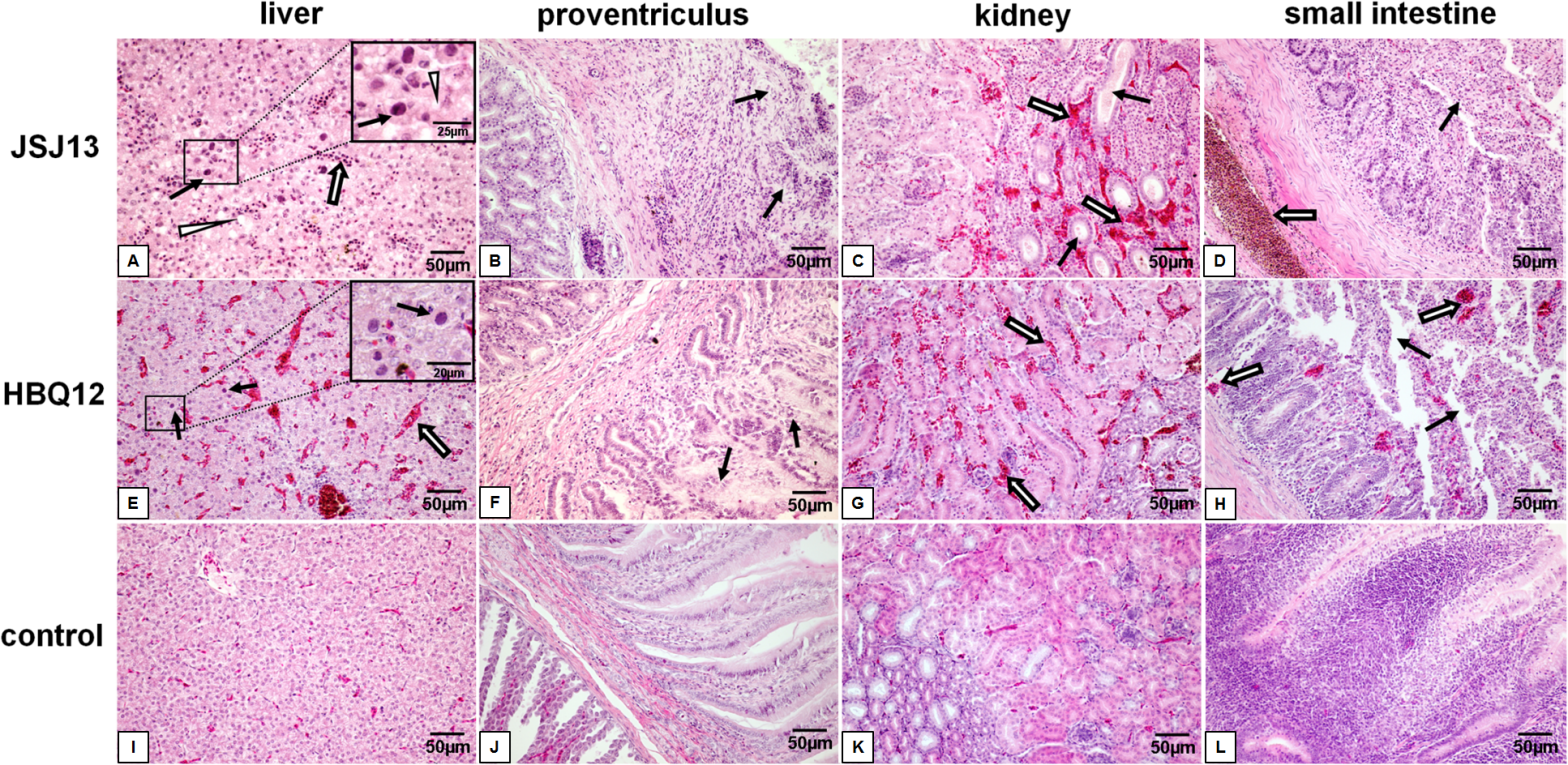
Histopathology in tissues from dead chickens infected with FAdV JSJ13 or HBQ12 (H&E) at 6 dpi. Liver: solid arrows indicate viral inclusion bodies in liver cells, open arrows indicate congestion in hepatic sinusoid and the triangles indicate hepatocyte steatosis. Proventriculus: solid arrows indicate dropout and necrosis of the mucosal epithelia in the proventriculus. Kidney: solid arrows indicate protein casts in renal tubules and open arrows indicate extensive congestion in renal interstitium. Small intestine: solid arrows indicate villus missing, drop out and necrosis of epithelial cells, open arrows indicate subserous congestion or dilatation and congestion of blood capillary in mucosa lamina proria. Scale bar = 50μm.

**Fig 6:**
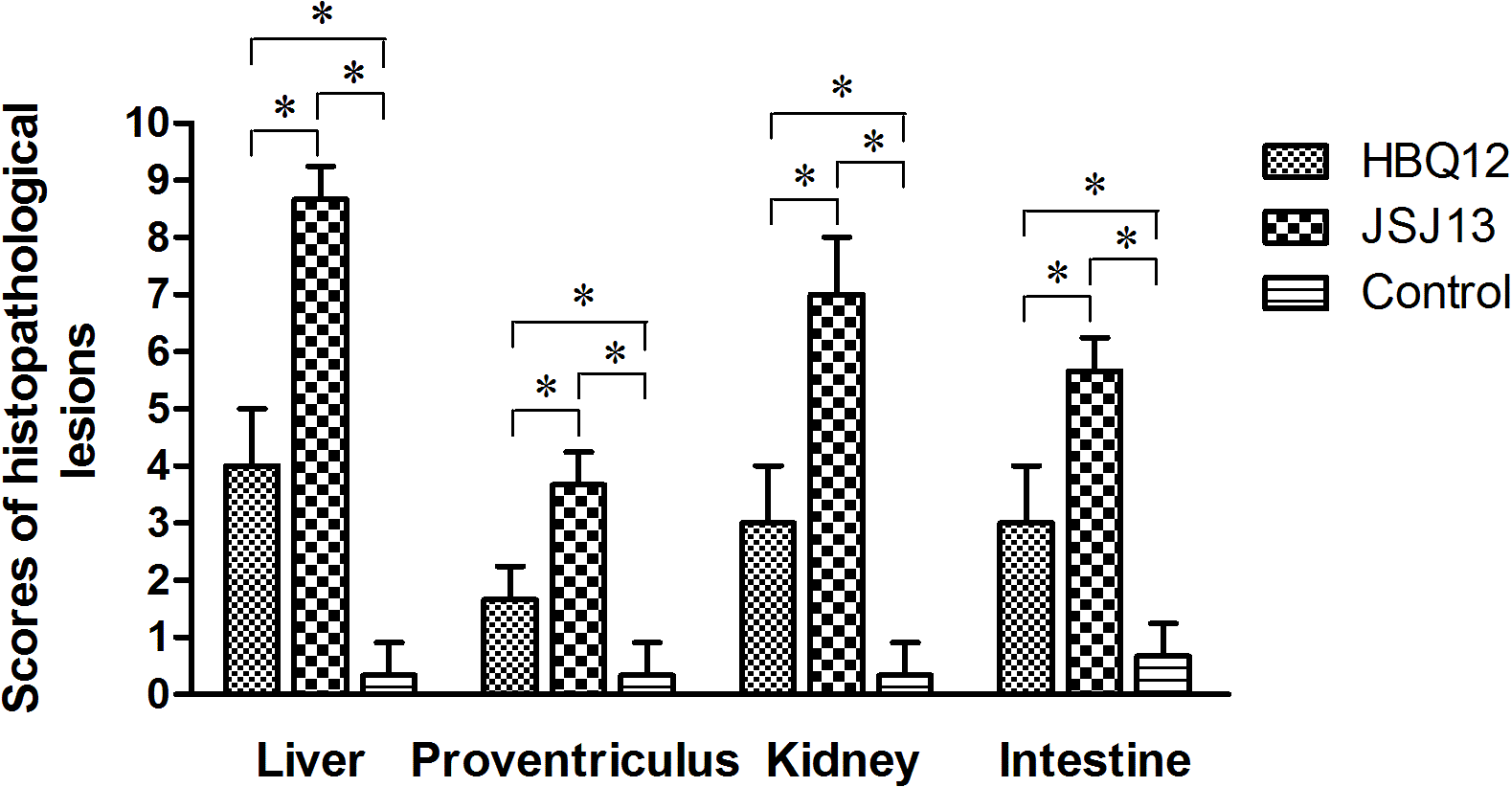
Scores of histopathological lesions in sampled tissues of chickens after inoculation with FAdV HBQ12 or JSJ13 strain at 6 dpi (n = 5 per group). Lesion scores: 0 for no lesion, 1–3 for mild lesions, 4–6 for morderate lesions and 7–10 for severe lesions. The mean lesion scores per group are shown. The error bars indicate standard deviations. Asterisks (*) mark significant differences between groups (P <0.05).

**Fig 7:**
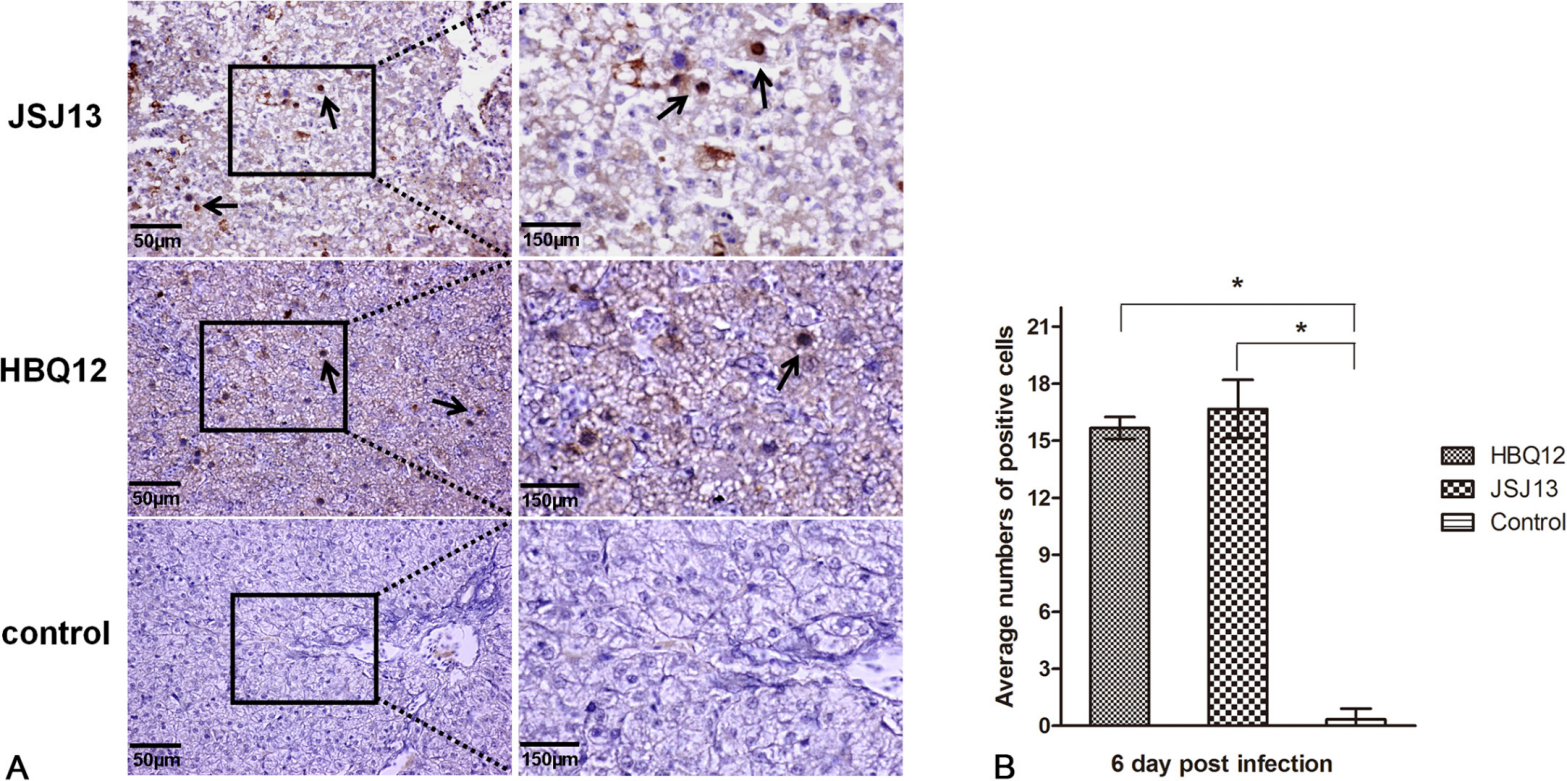
Immunohistochemical analysis. (A) Detection of FAdV antigens in liver tissues after infection with FAdV HBQ12 or JSJ13 strain at 6 dpi. Solid arrows indicate positive signals for viral antigen detected extensively in the liver cells. Scale bar = 50μm in left column figures and 150μm in right column ones. (B) A summary of the number of positive cells is shown, asterisks (*) mark significant differences between groups (P <0.05, n = 5). The error bars indicate standard deviation.

All chickens infected with HBQ12 or JSJ13 presented massive histological liver lesions with large fat vacuoles and necrotic focal areas, and basophilic intranuclear inclusion body could be seen in the hepatic cell as indicated by solid arrows (Fig 5A and 5E). Dropout and necrosis of the mucosal epithelia was observed in the proventriculus (Fig 5B and 5F). Protein casts in renal tubules and extensive congestion in renal interstitium were observed in the kidney (Fig 5C and 5G). The pathological changes in small intestine included the villus missing, drop out and necrosis of epithelial cells, subserous congestion or dilatation, as well as congestion of blood capillary in mucosa lamina propria (Fig 5D and 5H). No significant histologic lesions were present in the uninoculated control chickens (Fig 5I, 5J, 5K and 5L). Compared with the control group, the histopathological lesions of four tissues in infected group were obvious (P<0.05). The birds infected with JSJ13 caused greater histopathological scores than those of HBQ12 infection (P<0.05) (Fig 6). The positive signals for viral antigen were detected extensively in the liver cells by using immunohistochemical staining (Fig 7A). In two infection groups, a large number of positive cells were observed (P<0.05) on 6 dpi compared with the control group, but the difference was not significant between two infected groups (P>0.05) (Fig 7B).

### Viral DNA detection in tissues

Viral DNA in sampled tissues was detected by PCR and the results were summarized in Table 2. In group HBQ12, viral DNA could only be detected in liver and cecal tonsil collected at 3 and 5 dpi. Whereas total 29/36 (80.6%) of tissue samples collected from dead chickens possessed the detectable viral DNA. The similar results were also observed in group JSJ13, in which total 107/120 (89.2%) of tissue samples from dead chickens were positive for viral DNA, while at 3 and 5 dpi only liver and cecal tonsil in high ratio, occasionally spleen, kidney, pancreas and small intestine, possessed viral DNAratio ratio ratio. No viral DNA was detected in any tissues from chickens in the control group.

**Table 2:** Tissue distribution of two FAdV isolates in inoculated SPF chickens.^a^

Group	Birds	Detection in viral DNA from collected tissues^b^											
		Heart	Liver	Spleen	Lung	Kidney	Bursa	Cecum tonsil	Proventriculus	Small intestine	Pancreas	Thymus	Trachea
HBQ12	Killed (3dpi)	0/4	0/4	0/4	0/4	0/4	0/4	1/4	0/4	0/4	0/4	0/4	0/4
	Killed (5dpi)	0/4	1/4	0/4	0/4	0/4	0/4	2/4	0/4	0/4	0/4	0/4	0/4
	Dead	2/3	3/3	3/3	3/3	3/3	2/3	3/3	2/3	3/3	2/3	2/3	1/3
JSJ13	Killed (3dpi)	0/4	0/4	0/4	0/4	0/4	0/4	2/4	0/4	0/4	0/4	0/4	0/4
	Killed (5dpi)	0/4	4/4	1/4	0/4	1/4	0/4	3/4	0/4	2/4	1/4	0/4	0/4
	Dead	10/10	10/10	10/10	10/10	10/10	7/10	7/10	9/10	10/10	7/10	9/10	8/10
Control	Killed (3dpi)	0/4	0/4	0/4	0/4	0/4	0/4	0/4	0/4	0/4	0/4	0/4	0/4
	Killed (5dpi)	0/4	0/4	0/4	0/4	0/4	0/4	0/4	0/4	0/4	0/4	0/4	0/4

^a^Three-week-old SPF chickens were inoculated with FAdV HBQ12 or JSJ13 (10^3.5^ EID_50_/bird) via the oral route.

^b^Tissue samples from the euthanized birds at 3 and 5 dpi and dead chickens were examined by PCR method. Data are no. of positive samples/no. of tested samples.

### Antibody response

**Fig 8:**
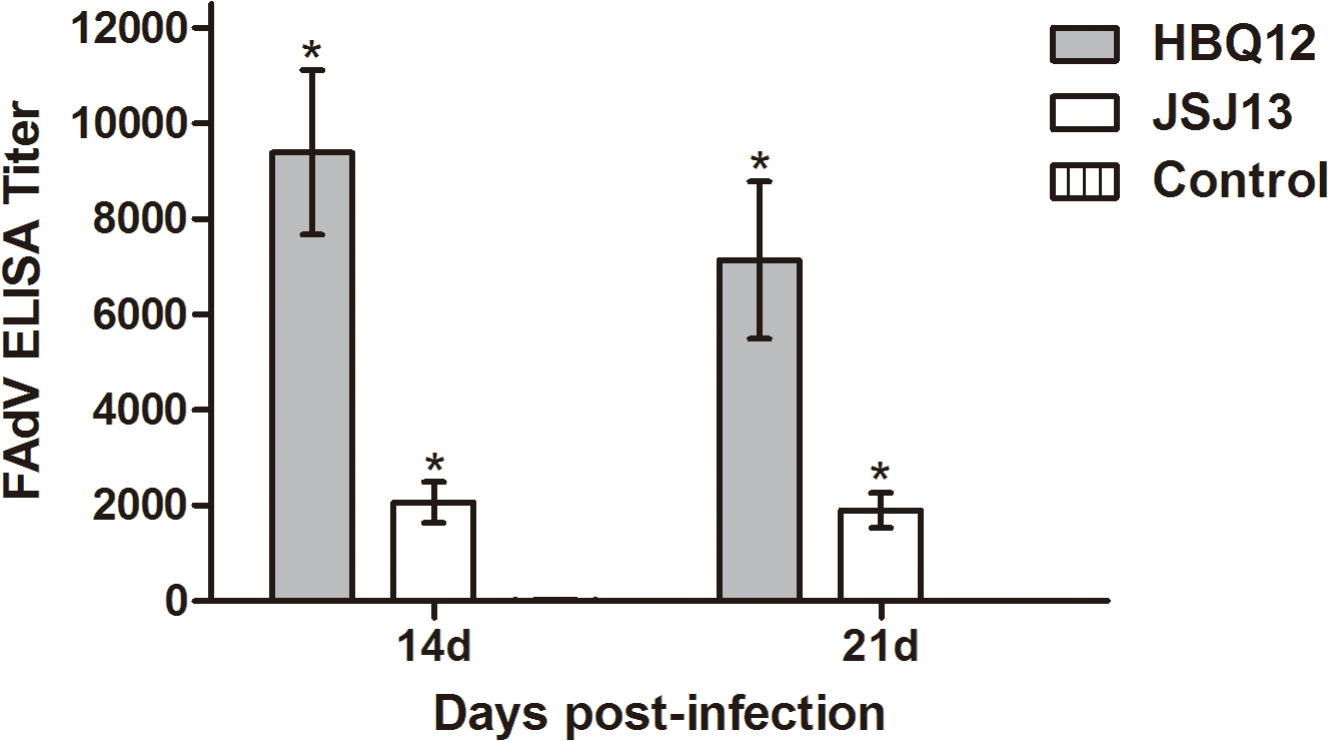
Antibody response to viral proteins in chickens inoculated with HBQ12 or JSJ13 by an oral route as measured by ELISA. Ten sera samples from each group were randomly collected at 14 and 21dpi. The grey and white bars represent HBQ12- and JSJ13-inoculated chickens, respectively. The bar representing the negative control group (vertical stripes) is not visible as antibodies were not detected in this group. The error bars correspond to 95% confidence intervals. Asterisks (*) mark significant differences between the mock infected group and the groups infected with virus.

The Ab response against two FAdV strains was shown in Fig 8. FAdV-specific Ab was not detected in any birds of the control group at all time points. Ab response to viral proteins appeared at 14 and 21 dpi with significant differences between inoculated groups and negative control (P<0.05). Chickens infected with strain HBQ12 had higher titers than those infected with strain JSJ13.

## Discussion

In recent years, increasing clinical cases of FAdV infections have been concerned and many FAdV strains related to IBH, HPS or GE have been isolated from most cases in many countries [7, 15–17]. Some of them are highly pathogenic to chickens and result in considerable economic losses [22–25]. In China, the outbreaks of IBH or HPS displayed an increasing trend since 2012. Most cases have been observed in broilers of 3 to 5 weeks of age, and occasionally in layers and breeder pullets aged 10 to 20 weeks. Mortality reported during these outbreaks varied from a slight increase to over 30% in severe cases and FAdV strains were isolated from all these cases. In the present study, three FAdV strains isolated from diseased chickens in China were phylogenetic and pathogenic analyzed. Among three FAdV isolates, strain HBQ12 and BJH13 were identified and classified as serotype 11 by sequence alignment and phylogenetic analysis, whereas strain JSJ13 was serotyped as FAdV-4. This is the first report of FAdV-4 strain circulating in China.

So far, the complete genome sequences available from all FAdV species were very limited: FAdV-A (1 strains, GenBank Accession No. U46933), FAdV-B (1 strain, GenBank Accession No. NC_021221), FAdV-C (2 strains, GenBank Accession No. HE608152 and GU188428), FAdV-D (1 strain, GenBank Accession No. AF083975) and FAdV-E (1 strain, GenBank Accession No. GU734104) [26–30]. A wide range of phylogenetic analysis in the whole genome levels would be very necessary and far-reaching for the deep understanding of the evolutionary relationship of aviadenovirus isolates. Given the whole genome of FAdVs haven’t been previously reported in China, the present study analyzed firstly the complete genome characterization of three Chinese FAdV prevalent strains. The complete genome of these FAdV isolates was 44,081, 43,966 and 43,756 bp, respectively, and possessed a similar organization with other aviadenoviruses. Both of HBQ12 and BJH13 belonged to FAdV-D and had reduced TR-1 and TR-2 sequence (3 identical repeats for TR-1 and 5 identical repeats for TR-2 in HBQ12, absent TR-1 and 5 identical repeats for TR-2 in BJH13), compared with other isolates that had been sequenced previously, which contained 5 identical repeats for TR-1 and 13 identical repeats for TR-2 [31]. As for strain JSJ13, no TR-1 or TR-2 sequence was found in the genome, which was consistent with sequenced KR-5 (GenBank Accession No. HE608152) and ON1 (GenBank Accession No. GU188428). However, the biological effects of these changes in virus genome are still unclear.

The pathogenicity of FAdV strains varied from subclinical infections to severe diseases [6, 10, 22–26, 30]. The pathogenicity of HBQ12 and JSJ13 strains were determined by observing the clinical signs, gross and histological lesions in chickens. The results demonstrated that both isolates had obvious pathogenicity in SPF chickens and led to 8.6% and 28.6% of mortality, respectively. Strain JSJ13 belonging to FAdV-4 showed higher pathogenicity in chickens than those of HBQ12 (FAdV-11) confirmed by clinical and histopathological scores. The results were consistent with several previous studies that parts of serotype 4 adenoviruses were highly pathogenic strains [5, 23, 25]. The varying degree of mortality correlates to the pathogenicity of the virus, different serotypes of infected strains and susceptibility of the chickens.

Most reported HPS were caused by FAdV serotype 4 (FAdV-4) belonging to FAdV-C, characterized by accumulation of transparent or straw-colored fluid in the pericardial sac, nephritis, and hepatitis with a high mortality of 30–70% [5, 9, 10, 23, 25]. In this study, obvious IBH and HPS were both observed in chickens infected with strain JSJ13 (FAdV-4) and HBQ12 (FAdV-11), suggesting that certain strains of other serotypes except for FAdV-4 are capable to induce HPS via oral route. What is more, maybe some strains of different serotype are able to reproduce IBH and HPS simultaneously.

In addition to the good management practices and strict biosecurity measures, the use of efficacious vaccines may help in prevention of FAdV infections. In some countries, inactivated oil-emulsion vaccine or new generation vaccine against FAdVs has been developed, and study has demonstrated that the inoculation of these vaccines is efficient to protect against IBH or HPS [9, 32]. Maternally derived antibodies to FAdV are known to protect chicks from infection, thereby the vaccination of breeders that produce an adequate level of maternally derived antibodies in progeny is an effective approach to protect young chicks from FAdV, prior to the development of age-related resistance [33]. The commercial vaccines against FAdVs have not yet been developed in China due to a lack of knowledge and understanding of the relevant diseases. Therefore, further genetic and pathogenic analysis of FAdVs is necessary and would provide useful information for the development of the efficient vaccine against IBH or HPS.

## Supporting Information

S1 Table Primers used to amplify the complete genomic sequence of FADV strain HBQ12 and BJH13. (DOC)

S2 Table Primers used to amplify the complete genomic sequence of FADV strain JSJ13. (DOC)
