# Correction: Pathogenicity and Complete Genome Characterization of Fowl Adenoviruses Isolated from Chickens Associated with Inclusion Body Hepatitis and Hydropericardium Syndrome in China

**DOI:** 10.1371/journal.pone.0161744

**Published:** 2016-08-22

**Authors:** Jing Zhao, Qi Zhong, Ye Zhao, Yan-xin Hu, Guo-zhong Zhang

The values in Table 1 and the corresponding values reported in the Sequence Alignment and Analysis subsection of the results are incorrect. The authors have provided a corrected Table 1 and article text here.

**Table 1:** Percent nucleotide sequence identities of the whole genomes of aviadenoviruses.

Species	Strain	Accession number	Homology		
			HBQ12	BJH13	JSJ13
FAdV-A	CELO	U46933	56.6	56.6	54.3
FAdV-B	340	NC_021221	63.4	63.3	55.4
FAdV-C	ON1	GU188428	54.9	54.8	**98.3**
FAdV-C	KR-5	HE608152	54.9	54.8	**98.4**
FAdV-D	A-2A	AF083975	**95.8**	**95.8**	55.2
FAdV-E	HG	GU734104	72.9	72.9	56.4
FAdV-D	HBQ12	KM096545	/	99.7	55.6
FAdV-D	BJH13	KM096546	99.7	/	55.5
FAdV-C	JSJ13	KM096544	55.6	55.5	/

## Sequence alignment and analysis

The percent sequence identity for available aviadenovirus whole genomes are given in Table 1. Strain HBQ12 and BJH13 were almost identical (99.7%) at the nucleotide level and they showed the highest sequence homology (95.8%) with strain A-2A (FAdV-D, Accession No. AF083975) isolated in the US at nucleotide level. Whereas they showed a low sequence identity (<72.9%) with the members of other aviadenovirus species. JSJ13 strain was more matched to the KR-5 strain (FAdV-C) isolated in Japan (Accession No. HE608152, 98.4% of identity at the nucleotide level). Sequence identities between JSJ 13 and other species ranged from 54.3% (between JSJ13 and FAdV-A) to 56.4% (between JSJ13 and FAdV-E).
