# Supplementary material for: Pathogenicity and Complete Genome Characterization of Fowl Adenoviruses Isolated from Chickens Associated with Inclusion Body Hepatitis and Hydropericardium Syndrome in China
Source: PLoS One. 2015 Jul 13;10(7):e0133073. doi: 10.1371/journal.pone.0133073 (PMC4500579; doi:10.1371/journal.pone.0133073)
Supplement: S1 Table — (DOC) [file pone.0133073.s001.doc]

**S1 Table. Primers used to amplify the complete genomic sequence of FADV strain HBQ12 and BJH13.^a^**

| Primer ^b^ | Location, bp | Upstream primer | Downstream primer | Length, bp |
| --- | --- | --- | --- | --- |
| 1 | 1~626 | CATCATCTATATATACCTACATGAA | GGACCAGGAGAGACGGATA | 626 |
| 2 | 438~1864 | TTGTGCGTTCTCCGTTGA | AATACCAGTCCCAGTAACCC | 1442 |
| 3 | 1505~2960 | TGTTTCTGTTTGGACCCTC | CATCACTCCCTCCTACGG | 1456 |
| 4 | 2730~4064 | CGCATGGATTTTGACTGT | ATGCGGAAGATCCATGTAT | 1335 |
| 5 | 3441~4650 | AAGATGTCAAGAGCGAGGTG | GACATAGAGCGGATAGCC | 1210 |
| 6 | 4021~5447 | TAAAGTAGGGCTCTGACCG | ACCCACCAACCCAAAGTA | 1457 |
| 7 | 5097~6474 | AAGGAGTCTTTTTGTTCGG | AGCAAAGAACTGGTCGTGT | 1338 |
| 8 | 5724~7096 | CGCAAGATAGAGTTGGTAA | AACAGGAAGACAGTCGGTA | 1373 |
| 9 | 6877~8168 | TTCCAAGCCGTTTGTTCC | CGCCTTCTTCAGCGAGTG | 1292 |
| 10 | 7764~9139 | CGTTCCAGCGTGTCGTAT | CCCAAATTCACTGCCAAA | 1376 |
| 11 | 8997~10442 | AGCGTGTCTAAGTGTTCC | ACAAGAGGGCTTCTACTGG | 1446 |
| 12 | 10227~17119 | AGAGGTTCGGTAGGTTTGTC | ATCTTTACTCGCACTTCGTC | 1493 |
| 13 | 11590~12818 | GATTGTTCGCTCCAGATGC | GACTGCTCGTACTCCGTCA | 1229 |
| 14 | 12378~13647 | GAAGAGCGTGAGCCAGTT | GCCTCCCTATCGTAATCGT | 1270 |
| 15 | 13310~14347 | CAGCATCAGCAGCAACAC | CTGGGTCAAACCGAACAT | 1038 |
| 16 | 13877~15404 | GGGATCTACTACCTGTACGACT | CCTTAGTTGACGCATCAGATA | 1528 |
| 17 | 15309~16670 | CTGCTTCAGAACAGACAGTCG | TGGTTCGGAAATTGCTGT | 1362 |
| 18 | 16537~17895 | GGGTGGAAGGAACAGCAT | TGAACCGTAGCCAGGGAC | 1359 |
| 19 | 17791~18934 | GGTGCTCCCGTTGAAGAA | CCGCGATGATGGGTATGA | 1144 |
| 20 | 18827~20151 | CGTGTTCAACGAGGAGAAG | GTGGTGACATTGCGAGTG | 1325 |
| 21 | 19749~21018 | GAAGAGGAGGAGAAAGAGGC | CACAGAGCGTAGTAGTGATGC | 1270 |
| 22 | 20714~22095 | CACAAACTATCTCGGAGTCAT | GCCGTCAGAGTAGGGAAT | 1382 |
| 23 | 21641~22824 | CCCAATATGATTCTACAGTCCA | CCGTAGTCCCAGCCATTA | 1182 |
| 24 | 22383~23762 | AGACCAACCAAGACGCTA | AGACCTCCGATAAAACCTG | 1380 |
| 25 | 23675~24854 | GCGTTCAACACTAATTCCG | CGCCTAAAACTACCTCCAA | 1180 |
| 26 | 24683~26113 | TCGGATTTCTTTTCGTCG | TGAGTCCGAAAGTAACGATCC | 1431 |
| 27 | 26037~27203 | GAGGAGCTGATTCTGATGCG | CGAGCACATAATCTTCCTTGTC | 1167 |
| 28 | 27032~28569 | GAGGTGCTCCATCATACTT | ACCCTCGTCAGCGTTATT | 1538 |
| 29 | 28254~29746 | CGACGAGAAGACCAGAGTAACC | CCACTTGGCTGGGTCCTT | 1493 |
| 30 | 29394~30926 | ATCAGACCAGCAACCCTT | CCGCTACTCCGATACCTT | 1533 |
| 31 | 30803~32378 | CCATTACCGCTGACCAAG | GTTTGATATGAGGAGAGTTCCCA | 1576 |
| 32 | 32089~33578 | GGCAGTAGTGACAAGGGAG | GGTAGATAGGCATCGCAAG | 1490 |
| 33 | 32492~33752 | TGCCTGTTCAGCACCATC | CCTGTATTCCGTAGTCAGTTGT | 1261 |
| 34 | 33703~34856 | ACTTTACCCTCACATACTTTGC | TTTGAGTAGGTCACCGTTTT | 1154 |
| 35 | 34747~36195 | AAACACACTGAGAACTCGTTACAC | CGAAGTTCCACGATGCGA | 1449 |
| 36 | 35993~37470 | ATCCGTATCATAGTACAACCG | CATAAAGAACAAAGTCCAGG | 1337 |
| 37 | 37353~38511 | TGTATAACGCTATGACTCACTG | GAGGGTCTCCACTTCTTTG | 1159 |
| 38 | 38270~38865 | GCCTACTCCTCAGCCTATC | CGCTCTATACCGTACCCAT | 596 |
| 39 | 38298~39667 | CCTGTGTTTCCCCGCCCGCTATAATACCC | GGTTTGGGGACAAGGTTTACACGGACGAG | 1370 |
| 40 | 39591~40983 | GACACCGCATTTACTTTCA | GCGTATGGCAGATGAGTC | 1393 |
| 41 | 40753~46132 | ATCCCTACGACTTAGTGACGCC | GTCTACCGTGTTCGTATTACAGCTT | 1380 |
| 42 | 41965~43283 | TCCTGTTGTAAGTCTAGCGTAT | GGGTAAGTGGGTCGTGAT | 1319 |
| 43 | 43160~44011 | GTGCTTAACGCTTCTTCTATG | CAGCAGAGACCGTGTGATAA | 852 |
| 44 | 43671~44081 | TAACCCTACCTTTCCATGAGT | CATCATCTATATATACCTACATGAA | 411 |

^a^ Primer locations are listed according to FAdV strain A-2A (FAdV-D, GenBank accession no. AF083975).

^b^ Primers were designed based on the available FAdV nucleotide sequences strain A-2A (FAdV-D, GenBank accession no. AF083975). All the primers were synthetized by Sangon Biotech (Shanghai, China).
