# Supplementary material for: Pathogenicity and Complete Genome Characterization of Fowl Adenoviruses Isolated from Chickens Associated with Inclusion Body Hepatitis and Hydropericardium Syndrome in China
Source: PLoS One. 2015 Jul 13;10(7):e0133073. doi: 10.1371/journal.pone.0133073 (PMC4500579; doi:10.1371/journal.pone.0133073)
Supplement: S2 Table — (DOC) [file pone.0133073.s002.doc]

**S2 Table. Primers used to amplify the complete genomic sequence of FADV strain JSJ13.^a^**

| Primer ^b^ | Location, bp | Upstream primer | Downstream primer | Length, bp |
| --- | --- | --- | --- | --- |
| 1 | 1~1213 | CATCATCTTATATAACCGCGTCT | CCTCCTCGGATCGTGTCAT | 1213 |
| 2 | 1150~2399 | TTGAGCGAATCTTTACACCG | TCCACCATAGTTCCCTCCC | 1250 |
| 3 | 2263~3620 | GTGGATTGGCGGAATAGGG | TTGGGTTGACGAAGTAAGAGCA | 1358 |
| 4 | 3387~4734 | GCGTCCTTCTTGATCCTCG | CGTCCACCTGTCCTGCTTC | 1351 |
| 5 | 4605~5979 | ACTTGTTCGTCTTCGGGTGTC | CCTGTTCCTCCAACTGCCTC | 1375 |
| 6 | 5859~7295 | GGATGCTACTCTGGCGTTGT | CGACTCCTTTCGCTGGTG | 1437 |
| 7 | 7114~8511 | GCGAGTCTGAGGGAGAAATG | CCACAACGAGCAGCTAACG | 1398 |
| 8 | 8415~9781 | GGGGTGTTCGGTGTCGTA | TAGACATCATCACGCTTCACAA | 1367 |
| 9 | 9593~10995 | GGGCGTTGCTGAGCATTT | CACCTTACCGTCCGATTTCTA | 1404 |
| 10 | 10705~12235 | ATGAAACGCACAAAGACGG | AGACAAGTCGGGAGACATCG | 1530 |
| 11 | 12044~13411 | GATGGTATCGCTGTTGGAAGTC | GTCACCGACAGATCCGGATTAC | 910 |
| 12 | 13174~14680 | TATCGCTGTTGGAAGTCGC | AGAGGAGTCGTCGTGGGTC | 1364 |
| 13 | 14383~15859 | TCTATACGTGCTTTCGGTGGT | GCTGCGGGTTCAGTTTGA | 1507 |
| 14 | 15617~16954 | TATCGCTCGGGACAGGTAGT | GCCGTAGTCGTAGAAGGTGC | 1477 |
| 15 | 16836~18132 | TTGCTCCGCTTGTTCGTG | CGGTAAGTGTCCCTTAATAATGG | 1338 |
| 16 | 17974~19310 | GCGGAATCAGAGGGTCGGGACT | ATCGGGCACCGTCAGCAAGG | 1297 |
| 17 | 18920~20333 | AACGCTGCTCCCCTTTTA | GCCCGTAGTCAGGTCTCG | 1337 |
| 18 | 20096~21856 | ACAGACAGGACGGACCAGC | TGCGAACCTAGACGAAACG | 1414 |
| 19 | 21567~23236 | GAGATGGTGACGGAGGTG | CCAGTTTCTGTGGTGGTTG | 1786 |
| 20 | 12044~13411 | CCAACGCCACTACCAACT | GAAAGCGGTGACGAGGAT | 1670 |
| 21 | 22906~24375 | GTGGACCATCCCGTTCAGT | GCATCGAGCAGTGCGTGT | 1470 |
| 22 | 24196~25465 | TGTGCGGGTGCTTGTGGT | GCGAGGTAGGAGGCGACTAA | 1270 |
| 23 | 25104~26537 | CTGGTCGTCTTCTTCTTCGG | CAGAGTCGCTAGAGTGGCTAAA | 1434 |
| 24 | 26362~27617 | CGGTTACTATTCGGCAGATGG | GATAAGCCTCGATGGTTTCCT | 1256 |
| 25 | 27372~28701 | CCTTCCATCACGGTTTCG | TGCTCATCTGGTCCTCTTCC | 1330 |
| 26 | 28610~29820 | GCCCGAAATCTACAATCCC | ACCTCCCATCATGCCTCC | 1210 |
| 27 | 29675~30942 | CAGACCAACAGCCCTACGC | CGAGCACTTTGAGCACCC | 1268 |
| 28 | 30804~32221 | GCCACTAAGCAAGCCAACG | CCTGATCCACGAGCAAGGT | 1418 |
| 29 | 32117~33234 | ACGATGACTGGGAACTGGC | GGACAAATGGACGATCAATAAA | 1118 |
| 30 | 33039~34380 | CCGCTACACCCTTCTATGCT | CGGTCCCTTCTGTGATTGC | 1342 |
| 31 | 34253~35709 | CGGAGATTTGCGATTGTGAGT | TGACTCATCATGGGTGTGGC | 1456 |
| 32 | 35553~37068 | ACACTAACTTCCTCATTGACCCTC | TGTCTGTCTGAACCTGCCTACC | 1512 |
| 33 | 36770~37942 | ACGATGGCGTGATAGGCGGAGC | ATGAACCGTAGCCCCGCCCTTT | 1173 |
| 34 | 37667~38921 | ACTACCGAGATCAGCCTGAAGA | CAGACTAAGGGAAAGTTGGAGAA | 1225 |
| 35 | 38750~40038 | GAAATGCTTCCTCCTTCACG | AAGTTTATAGGGATCTCGGGTTA | 1239 |
| 36 | 39930~41132 | AACCCGAGATCCCTATAAACTT | TAGTGCCTGTCCATTTGCC | 1203 |
| 37 | 40945~42236 | TGGCAAATGGACAGGCACT | TTGATTCGGTGGAGGTCGT | 1292 |
| 38 | 42047~43398 | CCCACTACCGCTACCACCAC | ATCACGCTGACGCTCCTCC | 1356 |
| 39 | 42817~43756 | AGCATGAATCAACTCGGTGTC | CATCATCTTATATAACCGCGTCT | 945 |

^a^ Primer locations are listed according to FAdV strain ON1 (FAdV-C, GenBank Accession No. GU188428).

^b^ Primers were designed based on the available FAdV nucleotide sequences strain ON1 (FAdV-C, GenBank Accession No. GU188428). All the primers were synthetized by Sangon Biotech (Shanghai, China).
